# Preparing for Climate Change: A Perspective from Local Public Health Officers in California

**DOI:** 10.1289/ehp.0800114

**Published:** 2008-12-08

**Authors:** Louise Bedsworth

**Affiliations:** Public Policy Institute of California, San Francisco, California, USA

**Keywords:** California, climate change, global warming, policy, public health

## Abstract

**Background:**

The most recent scientific findings show that even with significant emission reductions, some amount of climate change is likely inevitable. The magnitude of the climate changes will depend on future emissions and climate sensitivity. These changes will have local impacts, and a significant share of coping with these changes will fall on local governmental agencies. Public health is no exception, because local public health agencies are crucial providers of disease prevention, health care, and emergency preparedness services.

**Methods:**

This article presents the results of a survey of California’s local pubic health officers conducted between August and October 2007. The survey gauged health officers’ concerns about the public health impacts of climate change, programs in place that could help to mitigate these health effects, and information and resource needs for better coping with a changing climate.

**Results:**

The results of this survey show that most public health officers feel that climate change poses a serious threat to public health but that they do not feel well equipped in terms of either resources or information to cope with that threat. Nonetheless, public health agencies currently implement a number of programs that will help these agencies handle some of the challenges posed by a changing climate.

**Conclusions:**

Overall, the results suggest that local public health agencies in California are likely in a better position than they perceive to address the threats associated with climate change but that there is a larger role for them to play in climate policy.

Climate change is projected to increase average annual temperature in California by roughly 2–4°F by the middle of this century (Cayan et al. 2008c). By the end of the century, a medium-to-high emissions pathway is expected to increase temperatures by 4.5–10.5°F, whereas a lower emissions rate would keep the projected warming to 3–5.6°F (Cayan et al. 2008c). Since the start of the twentieth century, global average surface temperature has risen a little more than 0.3°F, with most of this increase occurring in the past 30 years (Cayan et al. 2008c). This increase in temperature is likely to result in accelerated sea level rise, increases in extreme heat events and in the frequency and severity of air pollution episodes. All of these changes will have direct and indirect impacts on public health.

Reducing emissions to lessen these impacts is necessary. Yet, the most recent analysis shows that even with aggressive emission reductions, some amount of climate change is inevitable (Cayan et al. 2008c; Intergovernmental Panel on Climate Change 2007). Therefore, in addition to efforts to lessen climate impacts, it will also be necessary to take steps to adapt to some amount of climate change. Although climate change is a global problem that requires global action to mitigate its risks, several studies have shown that the effects of climate change will vary spatially. Because of this spatial variation, the task of coping with the effects of climate change will fall heavily on local government agencies.

This is especially true in the public health sector, where local health agencies provide front-line health protection, disease prevention, and education. These impacts are likely to be more pronounced in several of the state’s most vulnerable populations, including the elderly, those living in poverty, and the infirm, populations often served by local health agencies.

California has adopted aggressive climate change policy goals. In 2005, Governor Schwarzenegger signed an executive order (Schwarzenegger 2005) establishing two greenhouse gas (GHG) emission reduction goals for the state, one for 2020 (reducing emissions to 1990 levels) and another for 2050 (reducing emissions 80% below 1990 levels). The first of these targets was codified into law through the California Global Warming Solutions Act of 2006. The executive order also stated that state agencies should prepare an assessment of the impacts of climate change on the state as well as an assessment of the state’s adaptation needs in the face of unavoidable climate change. An assessment of the impacts of climate change on the state was completed in 2005 (Cayan et al. 2008a, 2008b), and the second is under way. More recently, the state has undertaken the development of an adaptation strategy, which is being led by the California Natural Resources Agency (for more information, see California Energy Commission 2008). The adaptation strategy is assessing adaptation needs in several sectors, including biodiversity and habitat, infrastructure, water, oceans and coastal resources, working landscapes, and public health.

This study examined how local health agencies in California are prepared for dealing with a changing climate. The analysis is based primarily on a survey of local health officers throughout the state. California has 61 local health agencies, one in each county and in three cities (Berkeley, Long Beach, and Pasadena). Although the analysis focuses on California, these findings can provide lessons for other regions, as well.

This article begins with a background on the public health impacts of climate change. For each impact, I also discuss the primary coping or “adaptation” strategy that can be employed to lessen this risk. I then discuss the survey methodology, followed by a discussion of the survey results in three parts: perception of the risks of climate change, current readiness to cope with these risks, and resource and information needs. I conclude with a discussion of the implications of these results and next steps for the public health sector.

## The Climate Challenge for the Public Health Sector

Even under the most optimistic scenario, temperatures are expected to increase over this century. This increase in temperatures will have both direct and indirect impacts on public health (Ebi et al. 2006; Patz et al. 2005). The direct effect will be an increase in heat-related morbidity and mortality. In addition, the increase in temperatures could lead to increases in air pollution, changes in vector- and water-borne disease occurrence, and other issues that could pose risks to public health. The challenge to the public health community will be to respond to the general change in climate as well as to be prepared for potential increases in the incidence of extreme events such as heat waves or wildfires.

### Heat-related morbidity and mortality

Analysis of future climate change in California shows that even under an optimistic future emission reduction scenario, the incidence of extreme heat events is likely to increase by the end of the century (Drechsler et al. 2006; Hayhoe et al. 2004). Hayhoe et al. (2004) examined extreme heat occurrence in four locations in the state under both higher and lower emission scenarios. Table 1 summarizes their results.

The relationship between heat and mortality is location specific. Generally, regions that have higher average temperatures show a less dramatic relationship between heat and mortality (Drechsler et al. 2006). Under these scenarios, heat-related deaths in Los Angeles are predicted to increase 2- to 3-fold from a historic base of approximately 165/year under a low-emission scenario and up to 7-fold under a high-emission scenario by the end of the century (Hayhoe et al. 2004).

In July 2006, California endured an extended heat wave. Between 15 July and 1 August 2006, 140 deaths were classified as heat related by coroners and medical examiners. This is likely an underestimate because these deaths were of individuals found un-attended at home and do not include any delayed deaths. Most of the deaths occurred after the heat wave had been under way for several days, and 90% of the victims lived in socioeconomically depressed areas (where > 50% of the residents live below the federal poverty threshold) (Trent 2007). Roughly 65% of the deaths occurred in people ≥ 60 years of age (Trent 2007).

The elderly are one segment of the population that is most vulnerable to heat-related mortality. Other risk factors associated with extreme heat include the very young (<1 year of age), children < 5 years of age, chronic cardiovascular or respiratory disease, mental impairment, substance abuse, lack of air conditioning and heating, poverty, and living in urban areas (Basu and Samet 2002; Basu et al. 2008).

Extreme heat events also increase morbidity. Analysis of the 2006 heat wave in California discussed above showed a large increase in emergency department visits and hospital admissions during the event. The main reasons for emergency department visits were heat-related illness, electrolyte imbalance, acute renal failure, nephritis and nephritic syndrome, diabetes, and cardiovascular disease (Knowlton et al. 2009).

In addition to the risks associated with extreme heat events as discussed above, increases in average summer temperatures can also result in an increase in mortality. A recent analysis of temperature mortality in nine California counties between 1999 and 2003 showed that a 10°F increase in apparent temperature was associated with a 2.6–3.7% increase in mortality (Basu and Ostro 2008a, 2008b). These results indicate that even in the absence of extreme heat events like the 2006 heat wave, an increase in average ambient temperatures can result in an increase in mortality.

#### Primary adaptation measure

The most effective factor for protecting against heat-related mortality is having access to air conditioning (at home and in other locations) or cooling spaces (e.g., cooling centers such as malls or public buildings) as well as having access to transportation, living in a residence surrounded by trees and shrubs, being able to care for oneself, being physically active, and drinking extra fluids (Basu and Samet 2002). In California, lower income households are less likely to have air conditioning (Climate Change Public Health Impacts and Response Collaborative 2008).

The primary institutional adaptation measure to cope with extreme heat is a heat emergency plan and accompanying outreach and assistance for vulnerable populations. Analysis has shown that heat emergency plans can be effective in reducing mortality due to extreme heat (Ebi et al. 2004). Heat emergency plans are also implemented at the local level but involve coordination with state agencies and other nonprofit groups.

After the heat wave of 2006, the California Governor’s Office of Emergency Services (OES) issued a guidance report on the development of heat emergency plans (California Governor’s Office of Emergency Services 2006). Heat emergency plans tend to be phased plans that start with monitoring of heat indicators. As conditions warrant, additional phases are implemented.

Table 2 shows the heat emergency plan for San Diego County. This plan is in line with the OES recommendation, beginning with seasonal monitoring of heat indicators. The plan includes outreach to vulnerable populations, including a reverse 911 system to place calls to vulnerable populations to inform them of the risk of extreme heat and resources available to mitigate that risk. Bernard and McGeehin (2004) identify six central principles for a heat emergency plan, all of which are reflected in this plan (identification of lead and participating agencies; a consistent, standardized warning system; communication and public education; activities targeting high-risk populations; collection, and evaluation of information; and revision of the plan). In addition to reflecting these elements, the plan shown in Table 2 was revised using lessons learned from its implementation in 2005.

### Air pollution

Analysis of the effects of climate change on air pollution have shown that climate change is likely to lead to an increase in the severity and duration of air pollution episodes (Mickley 2007; Mickley et al. 2004). Air pollution levels can be affected by a number of direct and indirect effects of climate change. These include increased temperature, changes in biogenic emissions (e.g., emissions from vegetation), changes in chemical reaction rates, changes in atmospheric conditions that affect pollutant mixing, and changes in the atmospheric flows that affect pollutant transport (Hogrefe et al. 2004). In addition, behavioral responses to climate change could result in an increase in emissions, such as through the increased energy demand with higher temperatures (Franco and Sanstad 2008; Miller et al. 2008). There is also feedback between local air pollution and climate change, because some local air pollutants also have an effect on the climate.

The increase in atmospheric carbon dioxide concentration associated with climate change can also contribute to an increase in aeroal-lergens. For example, the amount of pollen produced by ragweed plants has been shown to increase with increasing carbon dioxide concentrations. Ragweed allergies can be particularly serious in people with asthma and other respiratory ailments (Knowlton et al. 2007).

#### Primary adaptation measures

Two primary adaptation measures deal with the effects of climate change on air pollution. The first is to modify emission reduction plans (e.g., regional air quality attainment plans and the state implementation plan) to account for the increase in air pollution attributable to climate change, the so-called “climate penalty” (Mickley 2007). This responsibility falls in the realm of air pollution control agencies at the federal, state, and regional level that are responsible for designing and implementing emission reduction programs. The second adaptation measure is to mitigate the health effects through public education and outreach programs to reduce emission-causing activities and limit exposure on days with high air pollution. Examples of such programs include the Bay Area Air Quality Management District Spare the Air program (2008) and the California’s Flex Your Power campaign (Efficiency Partnership 2008). Responsibilities for these programs can lie with local air quality and public health agencies as well as with state energy agencies and utilities.

### Infectious disease

Another indirect effect of climate change can be the incidence of infectious diseases. Changes in the climate can affect the range, incidence, and spread of infectious agents (Drechsler et al. 2006). Climate change will likely affect mosquito-borne diseases (e.g., malaria, dengue fever, and yellow fever) as well as those carried by ticks and other insects (e.g., Lyme disease) (McMichael et al. 2006). Weather influences the transport and dissemination of the microbial pathogens that can contaminate food and water and lead to illness. Changes in precipitation and runoff patterns could increase the risk of such contamination (Rose et al. 2001).

#### Primary adaptation measures

The primary adaptation measures for managing the spread of infectious disease are prevention programs that reduce vulnerability to infectious disease (e.g., avoiding exposure to mosquitoes), public education, and illness surveillance and tracking systems that can help to identify emergence of potential threats, and vector control, such as spraying for insect control.

Illness tracking and surveillance involve documenting patterns of disease among different groups of people. Such tracking can be used to detect the conditions that place populations at risk. Public health officials can then work to alter these conditions to reduce the population health risk (California Policy Research Center 2004). Illness tracking is currently conducted by local and state health agencies.

Only a few local health districts are responsible for vector control, but almost all areas of the state are included in a vector control program. These programs are operated by a number of different agencies around the state, including environmental health departments, mosquito abatement districts, and some cities.

### Wildfires

Climate change is expected to change the extent and characteristics of forests and other natural ecosystems and the risk of wildfires is expected to rise (Cayan et al. 2006; Westerling and Bryant 2006). Modeling that incorporates changes in temperature, precipitation, and simulated hydrologic variables estimates that the probability of large fires [> 200 hectares (494 acres)] in California could increase between 12% and 53% by the end of the century (Westerling and Bryant 2006). In addition to increased risks to property and infrastructure, wildfires pose a risk to human health. Forest fires result in increased concentrations of particulate matter, which have been linked to a number of adverse health outcomes, including cardiovascular disease, premature mortality, and asthma (Dockery et al. 1993). Because many wildfires occur in less inhabited areas, few air quality monitoring data show the effect of wildfires on ambient air quality.

#### Primary adaptation measure

The primary adaptation measures to decrease the occurrence and extent of wildfires lie primarily with other agencies, including state and federal forestry agencies that manage public lands, state and local authorities that oversee building codes and construction permitting, and state and local fire departments. Public health agencies have an important role to play in providing information to the public about the risks present during wildfires and actions that can be taken to reduce this risk. In addition, tools for outreach to vulnerable populations in other circumstances (e.g., extreme heat) could be used to reach vulnerable populations in the event of a wildfire.

## Methods

To understand how the risks posed by climate change are perceived by local public health officers in California and how prepared public health agencies are to manage the risks, I conducted a Web-based survey of public health officers around the state. An initial letter was sent to all 61 local health officers in the state, and then all future correspondence, including distribution of the survey, took place using electronic mail. The survey was distributed electronically in August 2007 and periodic reminders were sent via E-mail to all nonre-spondents. The survey remained available on the Web through the end of October 2007. In addition, I conducted interviews with a number of public health practitioners, including health officers, other government employees, and academics, to inform the creation of the survey and to provide context for the survey responses.

The survey was designed to answer four main questions: *a*) How large of a threat is climate change to public health, as perceived by local officials? *b*) What tools are in place that could help local public health agencies respond to the threat of climate change? *c*) Do local public health officials believe that they have adequate information and resources to respond to the public health threats associated with climate change? *d*) What information and resources are needed by local public health agencies to respond to the public health risks posed by a changing climate?

The first section of the survey asked questions about how large a risk climate change was perceived to be, what impacts of climate change were of concern, and whether the health agency had undertaken any programs specifically designed to address climate change. This section was followed by a series of question on actions undertaken to reduce the public health impacts of climate change, including what programs are in place and specific elements of those programs. The next section asked a series of questions on information and resource adequacy and what types of information and resources would be helpful to address climate change. The final section asked questions about policymaking. The complete survey can be found in Bedsworth (2008).

I received responses from 34 of the health officers, for a response rate of 56%. The jurisdictions that responded represented more than three-quarters of the state’s population and most regions of the state. The jurisdictions that responded included 7 of the 10 largest counties (by population) in the state and all but four of the state’s 16 counties with a population of more than 500,000.

## Results

The survey results provide insight into how local health officers perceive the risks posed by climate change as well as the current readiness of local health agencies to address these risks. They also provide insight into how prepared local health officers feel to manage the risks to public health posed by a changing climate. Each of these sets of findings is discussed below.

### Perception of climate change risks

I found wide agreement among local health officers that climate change poses a serious risk to public health: 94% believe that climate change is either a “very” or “somewhat” serious threat (Table 3). When asked to name the largest risk in their region related to climate change, public health officials most often mentioned extreme heat, followed by water-related issues, including supply, flooding, and risks to agriculture (Table 4).

Although the above results indicate a concern about the impact of climate change, most local health officers acknowledge that their agency has not yet undertaken programs specifically developed with climate change in mind. From the roughly one-quarter of agencies that have developed such programs, several officials mentioned heat emergency plans and working with local government on land-use planning issues. Other programs included encouraging carpooling and telecommuting, promoting hybrid electric vehicles, and raising climate change issues among the county board of supervisors.

**Figure 1:**
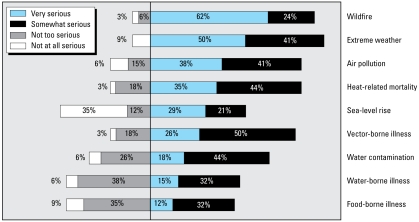
Perception of public health risks associated with climate change impacts among California health officials surveyed.

Respondents were provided with a list of health-related risks from climate change and asked to rank their seriousness (Figure 1). About 90% of respondents considered extreme weather to be either a “very” or “somewhat” serious threat to public health. Wildfire received the second-highest ranking, considered either a “very” or a “somewhat” serious risk by > 80% of respondents. This was closely followed by heat-related mortality, air pollution, and vector-borne illness, which were listed as either “very” or “somewhat” serious risks by more than three-fourths of the respondents.

Water- and food-borne illness, two areas that tend to be well under control, rank among the lowest levels of concern. Officials tend to be more concerned about those areas that we have less control over (e.g., extreme heat or wildfire) and where recent significant events have received substantial media attention (e.g., the 2006 heat wave and the 2007 Southern California wildfires).

Some of these results vary by location. Twenty-five of the 34 survey respondents are located in nonattainment areas in terms of the federal 8-hr ozone standards. Twenty-three (92%) of the respondents in these nonattainment areas indicated that air pollution was either a “very” or a “somewhat” serious risk posed by climate change. Among respondents in the nine areas that have attained the federal 8-hr ozone standard, only four (44%) listed air pollution as a “very” or “somewhat” serious risk from climate change, which was similar to the variation about sea-level rise. Eleven of the survey respondents are located in coastal areas. All of them listed sea-level rise as a “very” or “somewhat” serious risk posed by climate change. Among the 23 respondents located in inland areas, only 11 indicated that sea-level rise is a “very” or “somewhat” serious risk posed by climate change.

One result that does not show this type of variation is concern about heat-related mortality. Results show similar levels of concern between inland counties, which are typically warmer, and coastal counties. In both cases, about 80% of the officials listed heat-related mortality as a “very” or “somewhat” serious risk posed by climate change.

### Current readiness

The survey results show that local health agencies have several programs in place that could help them to confront the risks associated with climate change, although some updating will likely be needed.

#### Extreme heat

According to the survey, of the 34 jurisdictions that responded, 30 had a heat emergency plan in place. Of these plans, all include cooling centers and a process for identifying vulnerable populations. Almost 90% of the responding public health officers who have a heat emergency plan indicated that their programs monitor heat indicators, conduct public education, and include outreach to vulnerable populations. Local health agencies work with a number of other organizations to operate cooling centers as well as to provide other services (e.g., agricultural or domestic animal care). However, there are also some clear gaps. In particular, very few respondents to the survey indicated that their agency provided transportation to cooling centers (32%), and even fewer provided financial assistance to low-income residents to help with additional cooling costs (12%).

#### Air pollution

The survey responses indicate that about 62% of the local health agencies work with the local air district to publicize air quality information. In addition, almost 60% of respondents indicated that their agencies support programs designed to reduce either smog-forming or GHG emissions.

#### Infectious disease

Every jurisdiction that responded to the survey had a disease tracking program in place, although the diseases tracked varied. Most tracked mosquito-borne diseases, including West Nile virus (100%), western equine encephalitis (91%), and St. Louis encephalitis (91%). A little less than half of the respondents indicated that they tracked heat-related morbidity and mortality. Other diseases tracked by the respondents included asthma, cancer, and cardiovascular disease. All of these responses refer to surveillance that is occurring at the local level. The state, through the California Department of Public Health (CDPH), is also working to develop disease surveillance indicators for climate-change–related health effects, such as heat-related morbidity and mortality, air pollution, and vector-borne disease.

Approximately half of respondents indicated that their agency worked with the local vector control agency to identify areas for spraying and to publicize information about spraying activities. Almost all respondents indicated that their agency provided public education about vector control (94%).

#### Wildfire

When asked how serious a threat to public health was posed by a suite of climate impacts, 62% of the local health officers who responded to our survey indicated that wildfire poses very serious risk to human health. This was more than any other climate impact and was followed by extreme heat, which was indicated as a “very serious” risk by 50% of respondents. Although it is clearly a concern for local health officers, we have no comprehensive information on programs in place to address this risk. A review of the Web sites of local health agencies in areas affected by the wildfires in 2008 indicate that they provided information to the public on the health risks associated with wildfires and how to lessen exposure. The Monterey County Department of Health announced a public health advisory in response to the wild-fire in Big Sur. A public health advisory presents information on health risk, but may or may not require immediate action (the advisory is available at Monterey County Health Department 2008). The CDPH offered similar advice on lessening risk from exposure to the wildfires on its Web site.

### Resources and information

**Figure 2:**
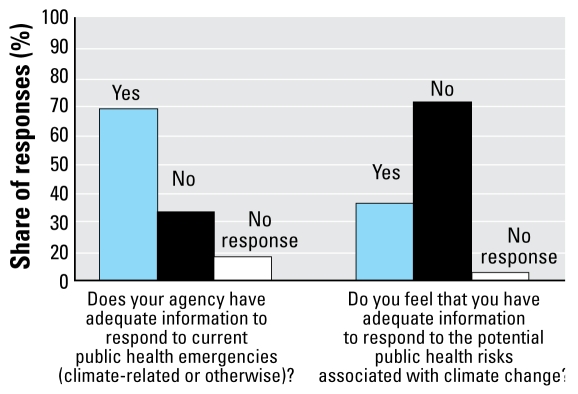
Information adequacy perceived among California health officials surveyed.

Most local health officers responded that they do not have enough information to respond to climate-related public health issues. This is particularly striking compared with whether they felt that they have enough information to respond to public health emergencies more generally (Figure 2). About two-thirds of the respondents indicated that they have enough information to respond to public health emergencies in general, but when asked whether they felt they had enough information to respond to climate-change–related public health emergencies, the results are almost the exact opposite.

**Figure 3:**
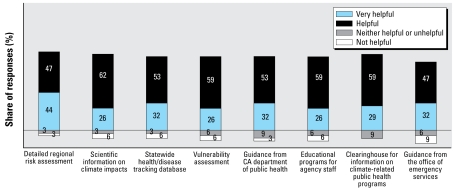
Value of information sources perceived among California health officials surveyed.

The desire for more information on climate risks became even more pronounced when respondents were asked what type of information would be helpful. Every option listed was believed to be either very helpful or helpful by at least 80% of the respondents (Figure 3). Detailed regional risk assessment of climate impacts received the largest share of “very helpful” rankings, at just more than 40%.

When asked who health officers would like to receive information from, almost 9 of 10 respondents indicated scientists (Table 5), followed closely by the CDPH (just more than three-quarters of respondents). These results agree with the findings on the types of information that respondents indicated that they would find most helpful (Figure 3). The highest rankings were for information that is most likely to come from the scientific community. This includes more detailed regional risk assessments (91% “helpful” or “very helpful”) and general scientific information on climate impacts (88% “helpful” or “very helpful”). The next highest ranked sources of information are likely to come from the CDPH. These include a statewide health/disease tracking database (85% “helpful” or “very helpful”), vulnerability assessment (85% “helpful” or “very helpful”), and guidance from CDPH (85% “helpful” or “very helpful”).

Other agencies that were indicated as preferred sources of information include the World Health Organization, the Centers for Disease Control and Prevention, and the National Institutes of Health.

Similar to their responses regarding availability of information, health officers indicated that they have inadequate resources to respond to the potential public health risks of climate change (68% yes, 15% no). When asked what resources they needed, roughly three-quarters of the survey respondents identified additional technical and analytical resources for health impact assessments (Table 6). This was followed closely by dedicated funding for climate-related activities.

## Discussion

Climate change will likely exacerbate a number of the issues currently being managed by the state’s public health institutions. This survey provides a first look at how well California’s local health agencies are prepared for handling a changing climate. Overall, the survey results indicate that local health officers feel that climate change poses a large risk to public health but that they lack the information and resources needed to address these risks. These findings on the perceived risks posed by climate change and lack of information and resources to manage these risks agree with findings from a similar survey of local health officers from around the country (Balbus et al. 2008).

Implementing adaptation measures in the public health sector will face a number of potential barriers. The first of these was clear in the survey findings: a lack of adequate resources. This is not a challenge that is unique to climate change, because public health agencies in California have long faced budgetary and resource constraints. Additional funding will be needed to adapt existing resources to best prepare the public health sector to manage the risks associated with climate change. This includes updating and refining heat emergency plans and including heat-related illness and other climate-change–related conditions in illness tracking programs.

State funding for public health programs is often linked to special funds or fees that restrict its use. Federal funding from the Centers for Disease Control and Prevention is linked to federal priorities. Therefore, in the absence of state or federal prioritization of climate change issues in the public health arena, funding resources are likely to remain a constraint to adopting adaptation measures.

A second challenge for addressing climate change in the public health arena will be the need to coordinate with other agencies in both the mitigation of and adaptation to climate change impacts. This type of coordination already occurs in many areas, such as vector control and air pollution control, but will become even more important under a changing climate. For example, mitigating the health risks associated with climate change will require outreach and education on the part of local health agencies, but state and federal forestry agencies will be responsible for reducing the risk of large wildfires occurring and posing an increased risk to public health. Such collaboration can be facilitated by CDPH and local health agencies taking on a larger role in California climate policy. This process is getting under way; the CDPH is the lead agency of developing the public health component of the state’s adaptation strategy.

Finally, a third challenge for public health agencies is to have access to practically oriented and actionable climate change information. California is far ahead of many other states in preparing information on the impacts of climate change on the state’s resources. But this information is not necessarily available in a form that allows public health agencies to design adequate adaptation strategies. For example, information on vulnerability to climate impacts will be needed at a community or even individual level for implementing effective heat emergency plans. Development of such information is just beginning to become available (see, e.g., Climate Change Public Health Impacts and Response Collaborative 2008).

Nonetheless, the survey does reveal some positive news. A number of the survey respondents have programs in place that can be helpful in responding to the public health risks associated with climate change. Although most of these programs were designed without climate change in mind and therefore will need refinement to be best suited to the climate challenge, the public health sector will not be starting from scratch as it addresses climate change.

## Figures and Tables

**Table 1:** Predicted changes in heat-wave days^a^ per year, mid- and late 21st century (Hayhoe et al. 2004).

		Midcentury (2020–2049)		End of century (2070–2099)	
Location	Baseline (1961–1990)	Lower emissions	Higher emissions	Lower emissions	Higher emissions
Los Angeles	12	24–28	35–36	44–47	76–95
Sacramento	58	91–93	101–104	109–115	134–138
Fresno	92	111–113	116–120	126–126	147–149
El Centro	162	176–185	180–185	149–162	178–204

a Temperature exceeds 32°C (89.6°F) for ≥ 3 days.

**Table 2:** Phases of San Diego County’s Excessive Heat Response Plan (County of San Diego Health and Human Services Agency 2006).

Phase	Description	Institutions
I. Seasonal readiness	Begin monitoring of heat indicators on a daily basis	Public Health Services Administration
	Announce opening and location of cooling centers, distribute fans and bus passes, if needed	Aging and Independence Services
	Develop and revise materials for agencies working with vulnerable populations	Office of Media and Public Affairs
	Convene Heat Plan Task Force	Emergency Medical Services
II. Increased readiness	Triggered by credible prediction of prolonged heat or power outages during warmer than normal conditions	Public Health Services Administration
	Release heat advisory press releases	Aging and Independence Services
	Monitor 911 calls, ambulance response, and emergency department visits and fatalities that indicate heat-related symptoms	Office of Media and Public Affairs
	Continue to monitor heat indicators	Public Health Services Emergency Medical Services Branch
	Notify all agency partners to provide outreach to vulnerable populations	
III. Heat alert	Triggered by excessive hot weather, night temperatures of ≥ 75°F for ≤ 3 days	Public Health Services Administration
	Continue public outreach	Aging and Independence Services
	National Weather Service advisories of excessive heat for ≤ 3 days, or high heat accompanied by blackouts	Public Health Services Emergency Medical Services Branch
	Enhance monitoring of 911 and other indicators and outreach to vulnerable populations	Office of Media and Public Affairs
	Institute daily calls among all involved agencies	
	Twice-daily check-ins with National Weather Service heat index	
IV. Heat emergency	Triggered by ≥ 3 days with a heat index^a^ >105°F, National Weather Service heat advisories or warnings for ≥ 3 days, abnormal medical emergencies and mortality due to extreme heat	Public Health Services Administration
	Issue regular media releases and brief public officials	Sherriff Department
	Consider declaring a public health emergency	Governor’s OES
	Activate Emergency Operation Center and Medical Operation Center	Public Health Services Emergency Medical Services Branch
	Send out Emergency Medical Alert Network notification to enrolled medical professionals and county staff	Aging and Independence Services
	Twice-daily check-ins with National Weather Service heat index	Office of Media and Public Affairs
	Enhance outreach to vulnerable populations and encourage cancelation of school-sponsored sporting events	
	Activate reverse 911 system^b^ to notify vulnerable populations	
	Continue to monitor 911 calls and other indicators and daily calls among all involved agencies	

a Determines how hot it feels based on temperature and relative humidity.

b Reverse 911 is a system that can place calls to populations to provide emergency information (phone numbers must be preentered).

**Table 3:** Perceived risks posed by climate change: “How large of a threat to public health do you feel climate change is?”

Answer	Percent
Very serious	56
Somewhat serious	38
Not too serious	3
Not at all serious	3

**Table 4:** Largest risk due to climate change: “What do you think is the largest risk to your region related to climate change?”

Answer	Percent^a^
Heat	35
Risks to agriculture	26
Water shortage	24
Flood	21
Wildfire	18
Human health	9
Water quality	6
Air quality	6
Habitat change	3
Sea-level rise	3
Economic vitality	3
No response	6

a Percentages calculated based on 34 respondents; 12 respondents provided more than one answer, for a total of 52 responses.

**Table 5:** Information sources: “If your agency would like more information on the public health impacts of climate change, who would you like your information from?”

Answer	Percent^a^	No.
Scientists	86	30
CDPH	77	27
California Air Resources Board	57	20
California Conference of Local Health Officers	57	20
Medical community	43	15
Other	6	2
No response	3	1

a Based on 34 respondents; respondents could indicate more than one choice.

**Table 6:** Resource needs: “What additional resources are needed to adequately respond to the potential public health risks of climate change?”

Answer	Percent^a^
Technical/analytical resources to assess health impact	96
Dedicated funding for climate activities	93
Staff with expertise in climate science	79
Technical/analytical resources to assess vulnerability	64
Better coordination with state agencies	43
Better coordination with local agencies	25
Other^b^	21
No response	21

a Based on 34 respondents; respondents could indicate more than one resource.

b In the “other” category, respondents indicated agencies with which coordination would be beneficial; these included the California Air Resources Board, CDPH, Department of Food and Agriculture, and OES.
